# Gait analysis of teenagers and young adults diagnosed with autism and severe verbal communication disorders

**DOI:** 10.3389/fnint.2013.00033

**Published:** 2013-05-20

**Authors:** Michael J. Weiss, Matthew F. Moran, Mary E. Parker, John T. Foley

**Affiliations:** ^1^Department of Psychology, Fairfield UniversityFairfield, CT, USA; ^2^Department of Physical Therapy and Human Movement Science, Sacred Heart UniversityFairfield, CT, USA; ^3^Department of Physical Therapy, Texas State UniversitySan Marcos, TX, USA; ^4^Physical Activity Laboratory, Department of Physical Education, State University of New York at CortlandCortland, NY, USA

**Keywords:** autism spectrum disorders, gait, motor control, verbal communication disorders, movement disorders

## Abstract

Both movement differences and disorders are common within autism spectrum disorders (ASD). These differences have wide and heterogeneous variability among different ages and sub-groups all diagnosed with ASD. Gait was studied in a more homogeneously identified group of nine teenagers and young adults who scored as “severe” in both measures of verbal communication and overall rating of Autism on the Childhood Autism Rating Scales (CARS). The ASD individuals were compared to a group of typically developing university undergraduates of similar ages. All participants walked a distance of 6-meters across a GAITRite (GR) electronic walkway for six trials. The ASD and comparison groups differed widely on many spatiotemporal aspects of gait including: step and stride length, foot positioning, cadence, velocity, step time, gait cycle time, swing time, stance time, and single and double support time. Moreover, the two groups differed in the percentage of the total gait cycle in each of these phases. The qualitative rating of “Body Use” on the CARS also indicated severe levels of unusual body movement for all of the ASD participants. These findings demonstrate that older teens and young adults with “severe” forms of Verbal Communication Impairments and Autism differ widely in their gait from typically developing individuals. The differences found in the current investigation are far more pronounced compared to previous findings with younger and/or less severely involved individuals diagnosed with ASD as compared to typically developing controls. As such, these data may be a useful anchor-point in understanding the trajectory of development of gait specifically and motor functions generally.

## Introduction

Movement disorders among individuals with an Autism Spectrum Disorder (ASD) have been gaining greater attention over recent years. Historically, movement disorders have been considered from two diagnostic perspectives. Primarily, forms of unusual movement have been characterized as one of the fundamental characteristics of ASD as a “narrow range of actions or interests” [Diagnostic and Statistical Manual of the American Psychiatric Association, 4th Edition—Revised, (DSM-4-R, 2000)]. Secondarily, “odd” movements have been described as an “associated feature” of ASD or in more extreme presentations a diagnosis of catatonia has been rendered (Wing and Shah, 2000, 2006). In either instance, little has been understood or studied in relation to *why* individuals diagnosed with ASD present with a wide array of differences in their movement and what relation these movement patterns may have to understanding the underlying etiology of the disorders.

The presentation of aberrant movements in ASD has been apparent from the first inception of the diagnosis (Kanner, 1943). Movement disorders have included a wide range of differences such as greater clumsiness, motor coordination abnormalities, postural control impairments and instability, hypotonia, muscle rigidity, akinesia, and bradykinesia, and more (Damasio and Maurer, 1978; Jones and Prior, 1985; Bauman, 1992; Kohen-Raz et al., 1992; Leary and Hill, 1996; Rogers et al., 1996; Rapin, 1997; Ghaziuddin and Butler, 1998; Molloy et al., 2003; Minshew et al., 2004; Donnellan et al., 2006, 2010). However, there is a growing number of researchers who have characterized disorders of movement as fundamental aspects of ASD (Leary and Hill, 1996; Donnellan et al., 2010; Fournier et al., 2010). This is a non-trivial distinction implying that differences in movement may offer clues to the underlying etiology of ASD, rather than simply being “associated” with the diagnosis.

The study of gait has been one domain of movement that has drawn interest for a number of years in this population. However, the relatively small numbers of empirical studies of gait that have been reported have varied in the methodologies and technologies used, participant ages, sample sizes, and ASD subtypes that have been studied (Vilensky et al., 1981; Hallett et al., 1993; Vernazza-Martin et al., 2005; Rinehart et al., 2006a,b; Calhoun et al., 2011; Esposito et al., 2011). Hence, it is not surprising that these reports have offered mixed findings in the extent and types of movement differences that have been found across these different individuals.

In considering some of the differing accounts of gait in this population, we are struck by two trends. First, every group of individuals diagnosed with an ASD who have participated in studies of gait show some form of movement differences as compared to typically developing control participants. This is consistent with Leary and Hill's (1996); Fournier et al.'s (2010) and Donnellan et al.'s (2010) similar conclusions that movement differences are pervasive among the entire population and as such should be thought of as a core deficit or difference in ASD.

Second, preliminary considerations indicate possible trends regarding the types of differences found in gait patterns correlating with the type of ASD that participants present with. Fournier et al. (2010) concluded that the pervasive differences in motor functions are not related to intelligence, to which we agree. However, there may be a correlation between the extent or type of differences found in gait as a function of the form or severity of the ASD diagnosis. By “severity” we are referring to the extent of difficulties in the so called “core deficits” of Autism—disorders or differences in communication, social interactions and range of actions and interests. Bear in mind that cognitive status has never been considered a “core deficit,” though ability to perform on any standardized cognitive test will co-vary with communication, social interaction and range of action skills (Zelazo et al., 1989; Zelazo and Weiss, 1990). Hence, we should be considering relations between the criteria of ASD such as the type of communication disorders a person presents with and movement patterns, rather than cognition, *per se*.

The few studies of gait that have been reported to date raise a question of whether a relation exists between types of movement differences shown by differing sub-groups of individuals on the Autism Spectrum and the extent of communication impairments. Vilensky et al. (1981) reported significant differences in a number of spatial and temporal parameters of gait between ASD and control participants. ASD participants in this study were described as having profound disorders of communication and social relatedness. Alternatively, Vernazza-Martin et al. (2005), whose participants' also presented with significant communication differences, found only relatively minor differences in spatiotemporal parameters of gait, *per se*. However, they found significant and meaningful variations or “oscillations” of the head, shoulders and trunk causing less stability and greater variability in posture as they walked. A series of other studies of individuals diagnosed with “High Functioning” Autism and/or Asperger Syndrome reported only minor variations in spatiotemporal parameters of gait, but reported significant variations in coordination, smoothness, consistency, and posture of the arms, head and trunk (Rinehart et al., 2006a,b), other parameters of posture and hypotonia associated with gait (Calhoun et al., 2011), or a generalized “clumsiness” among ASD participants as they walked (Hallett et al., 1993).

It is indeed likely that we will learn much from differentiating the gait patterns associated with differences among subtypes of ASD. Hence, it would be useful to segregate more precise descriptors of participants in the study of movement differences in those aspects of development associated with the diagnosis, such as specific descriptors of their social and language skills, or the types of narrow or repetitive range of actions and interests that these individuals show. Terms such as “high functioning” are routinely used in reference to cognitive status, which does not characterize the ASD diagnosis, *per se*. Similarly, the inclusion of an array of participants who share an ASD diagnosis, but have widely varied measures of communication, social or intellectual functions, needs to be differentiated if we are to tease out precise correlations with movement functions.

It is parenthetically interesting that Kern et al. (2010) demonstrated that the degree of “severity” in the ASD diagnosis has been shown to correlate with muscle strength. Similarly, Travers et al. (2012) found a correlation between ASD symptom severity and postural stability. These reports, coupled with the variations in reports of gait described above, indicate a need to differentiate the movement patterns of individuals who differ in their specific forms of ASD. Clearly, there is a need to unpack both the different aspects of movement that can be characterized, as well as clarifying the developmental presentations across the range of individuals who have an ASD diagnosis.

The intention of our current study was to evaluate gait patterns among a group of individuals diagnosed with ASD using narrowly defined *a priori* selection criteria of “severe” presentations of ASD in general and severe impairments in Verbal Communication specifically, among a group of older teenagers and young adults. We singled out the criteria of severe Verbal Communication impairments precisely because it is fundamental to the ASD diagnosis and because we wanted to look at the most extreme form of that criterion. We hypothesized that individuals with severe forms of Verbal Communication disorders would show widespread quantitative and qualitative aberrations in gait and other movement patterns reflected in CARS “body use” ratings, as compared to control participants of similar age- and gender.

## Materials and methods

Sacred Heart University's Institutional Review Board (IRB) approved all study protocols and procedures for this study.

### Participants

As shown in Table 1, nine participants with a prior diagnosis of autism (age range 16-years, 9-months to 22 years, 4-months of age, mean 19-years; one female and eight males) were recruited for the study. The Childhood Autism Rating Scale (CARS) was used to establish the appropriateness of the “severe autism” diagnosis for each participant, and that each participant presented with severe impairments of Verbal Communication. The CARS is a criterion-referenced diagnostic tool routinely used in the research literature (Perry et al., 2005; Mayes et al., 2009) as a standardized assessment of the degree of autism symptomatology across 15 developmental domains, e.g., “relating to people” and “object use.” Each domain is scored on a seven point Likert Scale with lower ratings, e.g., a score of 1 or 1.5 indicative of developmentally appropriate levels in each subscale, and high ratings, e.g., a score of 3.5 or 4 indicative of “severely abnormal” levels of each subscale. The subscales are then added together to form a “total score.” Scores from 15 to 30 indicate a “non-autistic” rating, 30–37 indicates a “mild-moderately autistic” rating, and 37 to 60 indicate a “severely autistic” rating. For the current study, the CARS ratings were performed by a psychologist experienced in developmental evaluations of the population diagnosed with Autism spectrum disorders. Following a standardized protocol, these ratings were done through observations of the potential participants and parental interviews within 1-month prior to participation in the study protocol.

**Table 1:** **Participants**.

	**Gender**	**Age (in years and months)**	**Height (inches)**	**Weight (pounds)**	**CARS total rating**	**CARS “Verbal Communication” subscale rating**	**CARS “Body Use” subscale rating**
**ASD PARTICIPANTS**							
E1	M	18-y, 7-m	75	195	51.5	4	4
E2	M	17-y, 3-m	71	199	49.5	4	4
E3	F	19-y, 1-m	69	148	52	4	4
E4	M	16-y, 11-m	70	205	59.5	4	4
E5	M	22-y, 4-m	70	240	41.5	4	4
E6	M	21-y, 6-m	71	162	48.5	4	4
E7	M	18-y, 3-m	70	172	50.5	4	4
E8	M	17-y, 6-m	73	170	59	4	4
E9	M	19-y, 10-m	70	165	48	4	4
ASD Group Means		19-y, 0-m	71.00	184.00	51.11	4	4
**CONTROL PARTICIPANTS**							
C1	F	19-y, 7-m	67	126	n/a	n/a	n/a
C2	F	19-y, 11-m	67	134	n/a	n/a	n/a
C3	F	20-y, 7-m	62	134.1	n/a	n/a	n/a
C4	M	19-y, 10-m	67	126.3	n/a	n/a	n/a
C5	M	16-y, 9-m	75	158.7	n/a	n/a	n/a
C6	M	20-y, 0-m	72	178.5	n/a	n/a	n/a
C7	M	19-y, 10-m	68	156.1	n/a	n/a	n/a
C8	M	20-y, 2-m	70	203.7	n/a	n/a	n/a
C9	M	19-y, 8-m	71	160	n/a	n/a	n/a
C10	M	20-y, 6-m	69.25	178.8	n/a	n/a	n/a
Control Group Means		19-y, 8-m	68.825	155.62			

Participants were selected to participate in this study if they met the following two criteria on the CARS: (1) a rating of “severely autistic” on their overall rating; and (2) at least a rating of 3 out of 4 on Sub-Scale XI “Verbal Communication,” which indicates a severe disorder of verbal behavior (i.e., not speaking in more than a few words or phrases; routinely not using verbally produced sentences as a principal mode of communication). As shown in Table 1, all of the experimental participants met the “severely autistic” criteria (mean ± *SD*; 51.11 ± 5.54), and all presented with severe disorders of Verbal Communication (indicated by a rating of “4” out of 4).

Ten control group participants of similar ages to the ASD participants (18–20 years of age, mean 19.5 ± 0.5 years; three females and seven males) were also recruited under IRB approval and with their consent. None of the participants in the control group had a known developmental or other health problem that would interfere with their performance. A series of *t*-tests revealed that the groups were not significantly different for age, height, and weight (Table 1). The CARS ratings were not conducted with the participants in the control group.

### Experimental protocol

The GAITRite (GR) Walkway System (CIR Systems Inc., Sparta, NJ) was used to collect spatiotemporal gait data. The GR is an electronic walkway (700 × 90 cm), with pressure sensors embedded in a horizontal grid. The recordable area of the mat is approximately 610 cm long × 61 cm wide. Sensors are separated at a distance of 1.27 cm, with a frequency of 80 Hz and temporal resolutions of 11 ms. The walkway is connected by a serial interface cable to a desktop computer running MS Windows XP.

For each individual trial, the participant walked along the length of the GR walkway. Participants completed six trials of preferred gait consecutively with about 30–60 s between each trial. Prior to the first trial, participants were given a demonstration and were then required to show their understanding of the instructions by walking down the mat. The participants were simply directed to walk to a research assistant who was standing approximately 2 m beyond the end of the GR mat. No participant required more than one demonstration and practice trial. The quantitative dependent variables collected via the GR Walkway System included both Spatial and Temporal measurements of gait and are described in Table 2.

**Table 2:** **Temporal and spatial measures of gait recorded by the GAITRite walkway system**.

**SPATIAL MEASURES**	
Step length (cm)	Measurement along the line of progression, from the heel center of the current footprint to the heel center of the previous footprint on the opposite foot.
Stride length (cm)	Measurement on the line of progression between the heel points of two consecutive footprints of the same foot (left to left, right to right).
Heel-to-heel base of support or base width (cm)	Vertical distance from heel center of one footprint to the line of progression formed by two footprints of the opposite foot.
Toe in/out (degrees from midline)	Angle between the line of progression and the midline of the footprint. The Toe in/out angle is zero if the geometric mid-line of the footprint is parallel to the line of progression; positive, toe-out, when the mid-line of the footprint is outside the line of progression and negative, toe-in, when inside the line of progression.
**TEMPORAL MEASURES**	
Cadence (steps/min)	Number of steps per minute across the walkway.
Gait cycle time (s)	Elapsed time between the first contacts of two consecutive footfalls of the same foot.
Velocity (cm/s)	Obtained after dividing the distance traveled by the Ambulation time.
Step time (s)	Time elapsed from first contact of one foot to first contact of the opposite foot.
Stride time (s)	Time elapsed between the first contacts of two consecutive footfalls of the same foot.
Heel contact (s)	Time that the first sensor appears in the heel quadrilateral of the foot.
Last contact (s)	Time that the last sensor goes off in any quadrilateral.
Toe off (s)	The time that the last sensor turns off in the forefoot quadrilateral of the foot.
Stance time (s) and percent of stance time (% of gait cycle)	The Stance Phase is the weight-bearing portion of each gait cycle. It is initiated by heel contact and ends with toe off of the same foot. It is the time elapsed between the first contact and the last contact of two consecutive footfalls on the same foot.
Swing time (s) and percent swing time (% of gait cycle)	Initiated with toe off and ends with heel strike. It is the time elapsed between the Last Contact of the current footfall to the First Contact of the next footfall on the same foot. It is expressed in seconds (s) and it is also presented as a percent of the Gait Cycle of the same foot. The Swing Time is equal to the Single Support time of the opposite foot.
Single support (s) and percent single support (% of Gait Cycle)	Time elapsed between the last contact of the current footfall to the first contact of the next footfall of the same foot. Single Support time is equal to the Swing Time of the opposite foot.
Initial double support (s) and percent initial double support (% of gait cycle)	The first period in the Gait Cycle in which both feet are on the floor. Initial double support occurs from heel contact of one footfall to toe-off of the opposite footfall.
Terminal double support (s) and percent terminal double support (% of gait cycle)	The second period in the Gait Cycle when both feet are on the floor. Terminal Double Support occurs from opposite footfall heel strike to support footfall toe-off.
Total double support (s) and percent total double support (% of gait cycle)	Total double support is the sum of the Initial added to the Terminal Double Support.

### Qualitative data collection

As indicated above, we used the CARS ratings to preselect participants as presenting with “severe” levels of global Autism and Verbal Communication only. We did not use any of the other sub-scales as criteria for inclusion in the study, other than how they contributed to the overall rating. That said, a reliable and valid qualitative index of movement abnormalities is included in the CARS on the sub-scale of sub-scale IV “Body Use.” This sub-scale is characterized by the authors (Schopler et al., 1988) as “representing both coordination and appropriateness of body movements. It includes such deviations as posturing, spinning, tapping, and rocking, toe-walking, and self-directed aggression… Consider such activities as cutting with scissors, drawing, or putting together puzzles in addition to active physical games. Evaluate the frequency and intensity of bizarre body use… ” (p. 13). Hence, the scale is a collection of aberrations in movement and actions. All of the sub-scales of the CARS are rated on a seven point scale from 1 to 4 (including “0.5” measures 1.5, 2.5, and 3.5). The characterizations for the Body Use ratings include a rating of:

1. *Age appropriate body use*—The child moves with the same ease, agility, and coordination of a normal child of the same age.
2. *Mildly abnormal body use*—Some minor peculiarities may be present, such as clumsiness, repetitive movements, poor coordination, or the rare appearance of more unusual movements.
3. *Moderately abnormal body use*—Behaviors that are clearly strange or unusual for a child of this age are noted. These may include strange finger movements, peculiar finger or body posturing, staring or picking at the body, self-directed aggression, rocking, spinning, finger-wiggling, or toe-walking.
4. *Severely abnormal body use*—Intense or frequent movements of the type listed above are signs of severely abnormal body use. These behaviors may persist despite attempts to discourage them or involve the child in other activities.

Where there was no pre-selection criteria used regarding the Body Use sub-scale, these ratings represented a valid qualitative dependent measure of each ASD participant's movement.

### Statistical analysis

All dependent measures were calculated as the average across the six repeated trials for each measure described above. Cadence and velocity were compared with Student's *t*-test's between the ASD and Control participants. All other analyses were performed as 2 (group) × 2 (Left and Right) Analysis of Variance (ANOVA).

## Results

### Homogeneity of variances

All of the following parameters were assessed to determine if the variances in each between group analyses were homogeneous, using Levene's Test of Equality of Error Variances. Only two of the following analyses were found to have significant group differences in their respective variances—the analyses of Double Support Load Time and Double Support Unload Time (*F*-values are presented with those analyses below). No other analyses revealed a statistical lack of homogeneity of variances.

### Spatial parameters

As shown in Table 3, a number of spatial parameters differentiated the ASD and control participants. Step length was longer for the control's (*F* = 7.12, *p* < 0.016), with no differences between Left or Right legs or Group × Leg (Left vs. Right) interactions. Similarly, the groups also differed in Stride Length, with the Controls being significantly longer than those in the ASD group (*F* = 6.72; *p* < 0.019), with no difference found in Left vs. Right sides or Group by Leg interactions.

**Table 3:** **Summary of ASD and control participants' means (standard deviations) and *p*-values compared on spatial and temporal parameters of the gait cycle**.

	**Control group**	**ASD group**	***p*-value <**
**SPATIAL PARAMETERS**			
Step length left (cm)	78.13 (5.47)	67.96 (9.18)	0.016
Step length right (cm)	78.265 (7.07)	69.93 (8.68)	0.016
Stride length left (cm)	156.5 (12.22)	139.09 (17.16)	0.019
Stride length right (cm)	156.44 (12.2)	138.12 (18.17)	0.019
Support base left (cm)	10.74 (1.96)	10.04 (1.98)	n.s.
Support base right (cm)	9.94 (1.7)	9.75 (1.74)	n.s.
Toe in/out angle left (degree)	0.87 (4.82)	14.67 (11.93)	0.002
Toe in/out angle right (degree)	4.02 (3.39)	15.03 (8.95)	0.002
**TEMPORAL PARAMETERS**			
Cadence (steps/min)	112.52 (5.02)	100.11 (11.18)	0.0055
Cycle time (s)	1.07 (0.025)	1.22 (0.0065)	0.004
Velocity (cm/s)	146.5 (9.81)	116.11 (26.66)	0.009
Step time left (s)	0.5347 (0.02)	0.6142 (0.07)	0.004
Step time right (s)	0.5323 (0.03)	0.6017 (0.06)	0.004
Left stance (s)	0.659 (0.03)	0.783 (0.1)	0.001
Right stance (s)	0.663 (0.03)	0.785 (0.1)	0.001
Left swing (s)	0.41 (0.02)	0.427 (0.04)	0.1
Right swing (s)	0.40 (0.03)	0.43 (0.04)	0.1
Single support left (s)	0.40 (0.03)	0.43 (0.04)	0.1
Single support right (s)	0.41 (0.02)	0.427 (0.04)	0.1
Heel off/on left (s)	0.1697 (0.09)	0.1242 (0.1)	n.s.
Heel off/on right (s)	0.1667 (0.07)	0.1305 (0.1)	n.s.
Double support left (s)	0.2569 (0.02)	0.3479 (0.07)	0.001
Double support right (s)	0.2565 (0.02)	0.344 (0.06)	0.001
Double support load left (s)	0.1288 (0.01)	0.1684 (0.03)	0.001
Double support load right (s)	0.129 (0.01)	0.1799 (0.04)	0.001
Double support unload left (s)	0.1282 (0.01)	0.1797 (0.04)	0.001
Double support unload right (s)	0.1292 (0.01)	0.1642 (0.03)	0.001

**Figure 1:**
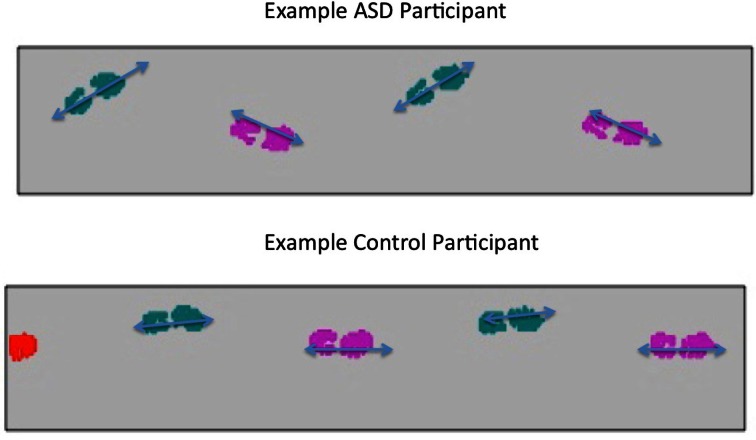
**Example of foot positioning of ASD and Control Group participants**.

The two groups also differed in the extent to which their feet positions varied, indexed by the toes pointing In or Out. As exemplified in Figure 1, the ASD group showed a positive index, indicating that their Left foot was pointed outward from the line of progression by 14.67° and right foot by 15.03°, compared to the control participants who showed an average of 0.87° on the left and 4.02° on the right feet (*F* = 13.94, *p* < 0.002). Though the orientation of the feet implied a subtly wider base of support for the ASD participants, there was no significant difference found in the Heel-to-Heel Base of Support between the two groups (*F* = 0.31, *p* < 0.59). Again, no differences were found in any of these analyses in Left vs. Right Leg or a Group by Leg interaction.

### Temporal parameters

Widespread temporal gait differences were found between groups (Table 3). The participants in the control group walked with a greater Cadence [*t* = 3.18 (*df* = 17), *p* < 0.0055], Velocity [*t* = 3.23 (*df* = 17), *p* < 0.009], and Gait Cycle Time (*F* = 11.29, *p* < 0.004) as compared to the ASD group. The ASD participants also showed longer time durations for their step time (*F* = 11.26, *p* < 0.004) and stance phase (*F* = 14.37, *p* < 0.001). There was also a trend for a significantly longer swing phase for the ASD participants compared to controls (*F* = 2.94, *p* < 0.1), with a correspondingly identical trend for Single Support Time (*F* = 2.94, *p* < 0.1; which is occurring concurrently with the opposite Leg Swing Times). No significant differences were found in any of these analyses for Left vs. Right Leg Differences or Group × Leg interactions. There were also no differences found in the duration of Heel Off and On for either Leg or Group (*F* = 0.901, *p* < 0.36).

The groups also differed with the ASD participants having both feet on the ground simultaneously as indicated by Initial Double Support time (*F* = 16.48, *p* < 0.001), Terminal Double Support time (*F* = 16.56, *p* < 0.001) and the corresponding Total Double Support time (*F* = 17.09, *p* < 0.001). An analysis with Levene's Test of Equality of Error Variances did reveal group differences in the homogeneity of variance for both the Initial [left leg (*F* = 4.36, *p* < 0.052) and right leg (*F* = 5.53, *p* < 0.031)] and Terminal Double Support [left leg (*F* = 6.37, *p* < 0.022) and right leg (*F* = 8.38, *p* < 0.01)]. As stated above, these were the only analyses that revealed a lack of homogeneous variances across all other tests.

### Temporal parameters: percentage of gait cycle

**Figure 2:**
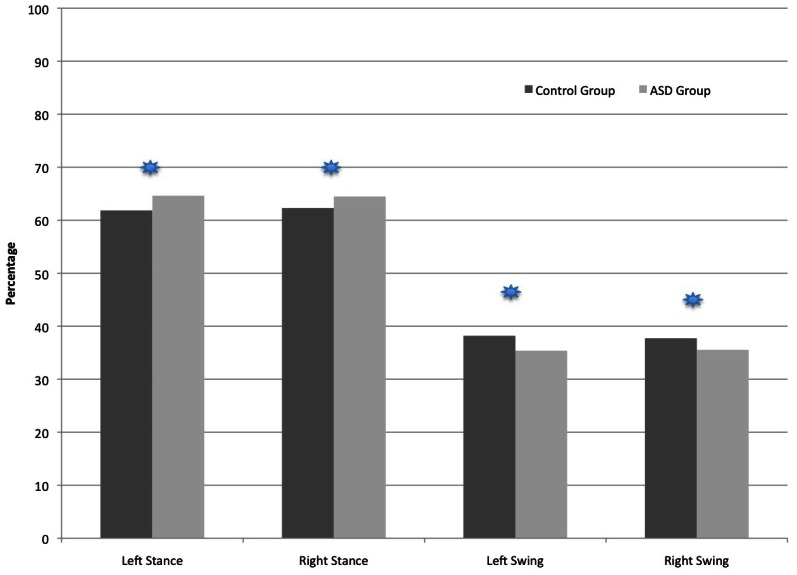
**Comparison of the two groups in the percentage of time spent in the Stance and Swing Phases of the Gait Cycle.** *p* < 0.001 (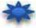).

**Figure 3:**
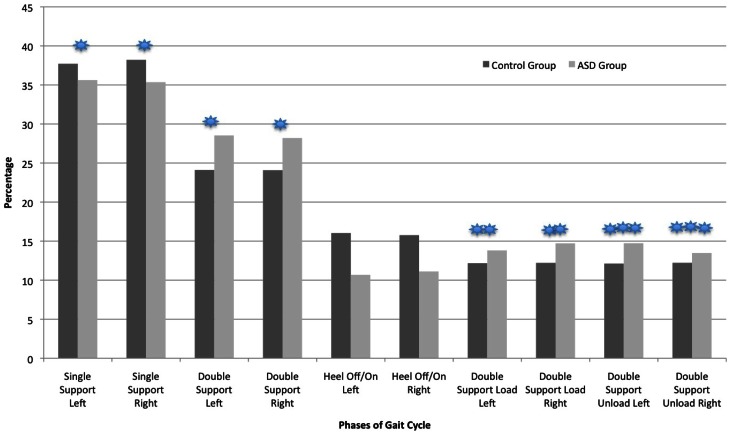
**Comparison of the two groups in the percentage of time spent in each Gait Cycle phase.** *p* < 0.001 (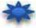); *p* < 0.003 (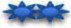); *p* < 0.004 (
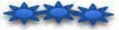
).

As shown in Figure 2, the ASD participants spent a greater percentage of the total Gait Cycle in the Stance phases and less time in the Swing phase relative to controls. The percentage of Swing Time (and corresponding alternate leg Single Support percentage shown on Figure 3) was larger for the control compared to ASD participants (*F* = 14.99, *p* < 0.001). Alternatively, the percentage of Stance Time was larger for the ASD participants (*F* = 14.95, *p* < 0.001). Figure 3 shows the distribution of Gait Cycle elements, which differ between groups on all but one element across the entire cycle. Each element that contributes to Total Stance time differs between groups in the percentage of time spent in Initial Double Support Load (*F* = 11.96, *p* < 0.003), Terminal Double Support Unload (*F* = 11.45, *p* < 0.004) and Total Double Support (*F* = 14.8, *p* < 0.001). There were no differences found in the percentages of Single Support, Stance Time or Double Support elements comparing Left vs. Right Legs or Group × Leg interactions. The only percentage of the Gait Cycle in which between group difference were not found was in the percentage of Heel Contact (*F* = 1.78, *p* < 0.2).

### Qualitative presentation of movement: cars ratings of “body use”

All nine participants in this study diagnosed with ASD showed the highest rating—4 out of 4—on the sub-scale of sub-scale IV “Body Use” (see Table 1). These ratings were predicated upon an array of unusual and otherwise inappropriate actions demonstrated by each of the ASD participants such as finger waving or contorting, hand and/or arm flapping, halting or ballistic movements of the hands, arms or legs, seemingly uncontrolled rocking or jumping movements, a “skipping” gait, stilted, stiff or “freezing” body postures, repetitive actions such as touching or poking at objects, various unusual facial contortions, peculiar grasp patterns, difficulty either moving from a standing to a seated position or visa-versa, and more.

## Discussion

### Summary of findings

In this study, we found that older teenagers and young adults diagnosed with globally “severe” forms of ASD that included severe ratings of Verbal Communication disorders, quantitatively walked slower, taking shorter steps, are in a stance position longer and swing their limbs for a shorter percentage of their spontaneous gait cycle. Though there is not a difference found in the Groups' Bases of Support *per se*, individuals with ASD show a significant variance in their foot positions relative to Controls vis-à-vis Toe Out positioning relative to their gait line of progression.

Though these individuals were selected to participate in this study due to both global ratings of the severity of their Autism and severity of their Verbal Communication, participants were also rated to engage in the most severe forms of movement abnormalities as indexed by the CARS rating scale. Hence, as we predicted individuals who have severe forms of Autism and low Verbal Communication will also have significant variations in gait and other qualitative aspects of their movement.

These findings are consistent with a number of studies that report movement differences between individuals diagnosed with ASD as compared to typically developing individuals. However, a number of differences also exist between our study sample, and their associated results, compared to other samples previously reported (cf., Fournier et al., 2010), which will be considered below.

### Is there a relation between differences of movement and level of “severity” of ASD?

In reviewing the range of studies that have been reported to date there are some meaningful differences in gait that may correlate with the severity of the ASD diagnosis. All of the studies report differences between individuals diagnosed with ASD as compared to typically developing comparison groups. However, these differences are reflected in different parameters related to gait. In our current study, all of the participants presented with severe forms of the ASD diagnosis and showed widespread differences in both quantitative and qualitative aspects of gait and movement, respectively. Our findings are in contrast to the data reported by Rinehart et al. (2006a,b); Calhoun et al. (2011), and Hallett et al. (1993) who reported only limited or no differences in spatiotemporal parameters of gait among individuals diagnosed with “High Functioning” forms of ASD. Rinehart et al. reported that young children diagnosed with High Functioning Autism (HFA) and Asperger Syndrome (AS) showed greater *variability* in stride length, though the average stride length was comparable to that of the typically developing children. They did find meaningful differences between groups in qualitative indexes of movement (e.g., upper body postural variations, smoothness of movement, etc.), but little in the way of quantitative differences in temporal and spatial parameters of gait, *per se*.

Similarly, Calhoun et al. (2011) reported data from “high functioning” children (mean age of 6.3-years) in which they found that the ASD individuals had a significantly higher cadence compared to controls, but there were few other temporal and spatial parameters of gait that differentiated the ASD from control participants. For example, Calhoun et al., like Rinehart et al. found no significant differences in stride length, or in the percentage of the gait cycle time spent in the stance phase. However, Calhoun et al. (2011) found widespread and significant differences between an Autism group compared to typically developing children (mean age of 6.3-years) in peak hip and ankle kinematics and kinetics. Significant differences were found for sagittal ankle and hip components, indicative of reduced plantarflexor moments and increased dorsiflexion angles, which may be associated with hypotonia. Furthermore, decreased hip extensor moments were found for the autism group compared to the control group. Indeed, independent clinical evaluation of the ASD participants in that study resulted in 33% of the group being diagnosed with hypotonia and gross motor delays were reported in 25% of the participants.

The only prior data regarding adults that utilized three dimensional kinematic data acquisition with synchronously processed kinetic information (force plate data) was reported by Hallett et al. (1993). Participants in that study were 25 to 38-years of age and described as “high functioning and had good language ability” and were reported to have Wechsler Adult Intelligence Scale-Revised (WAIS-R) full-scale scores ranging from 78 to 107 (mean of 88, SD ± 12). Though the authors reported “mild clumsiness” in four of five ASD participants and differences in upper limb posturing during gait in three of the five participants, there were few specific aspects of gait that the groups differed on as compared to typically developing age-matched adults. The velocity of gait, step length, cadence, step width, stance time, and vertical ground reaction forces were comparable to the control participants. The ASD participants did show decreased range of motion of the ankle and decreased knee flexion in early stance reminiscent of the data reported subsequently by Calhoun et al. (2011). Moreover, their “awkwardness” was similar to the qualitative findings reported by Rinehart et al. (2006a,b).

Alternatively, Vilensky et al. (1981)—similar to the data that we report here—found variations between their ASD group compared to typically developing age-matched controls on temporal and spatial elements such as reduced stride lengths and increased stance times not found by Rinehart et al. (2006a,b); Calhoun et al. (2011), or Hallett et al. (1993). Vilensky et al. did report increased hip flexion at toe-off, and decreased knee extension and ankle dorsiflexion at ground contact, all similar to data reported by Calhoun et al. and consistent with the qualitative ratings reported by Rinehart et al. Also related to level of function, Vilensky et al. (1981) reported a significant negative correlation between level of Intelligence and the ankle joint angle at initial contact with the floor. Hence the authors concluded, “Thus the more intelligent children had heel strikes that more closely resembled those of the normal children.”

The one exception that we have found in relation to severity of ASD and type of movement anomalies was reported by Vernazza-Martin et al. (2005). These authors characterized their study group as presenting with “pronounced alteration of social interactions, a lack of verbal communication (p. 93),” and children diagnosed with Asperger Syndrome were excluded from the study. These authors found only relatively minor differences in spatiotemporal parameters of gait between age-matched 4-to-6-year old children diagnosed with ASD as compared to typically developing controls. However, these authors report significant “oscillations” of the head, shoulders and trunk stability among the children diagnosed with ASD. These findings indicated meaningfully reduced stability and greater variability in posture as they walked similar to Rinehart et al. (2006a,b) and Calhoun et al. (2011), despite the fact that the participants are seemingly “lower” functioning compared to these other reports.

Finally, any consideration of a possible relation between level of function and aspects of gait also must include the findings reported by Kern et al. (2010) who demonstrated that the degree of “severity” in the ASD diagnosis correlates with muscle strength. Similarly, Travers et al. (2012) found a correlation between ASD symptom severity and postural stability.

Though we clearly need to be cautious in any direct comparison from our data to those reported above, we find it informative to consider the possibility that there is a relation between the level of severity in the ASD diagnosis of the participants and their corresponding characteristics of movement. Those studies reporting data from “high functioning” participants showed only mild variations in their temporal and spatial gait patterns, with more prevalent differences in the smoothness of movement and postural controls. There are unmistakable differences in the movement patterns of these individuals. However, their findings are in marked contrast to our report of severe levels of functioning in our population, who also show far more significant variations in the temporal and spatial gait patterns as compared to the control participants.

When these few studies are taken together, they beg the question for further study to address the relation between severity of the ASD diagnosis and movement aberrations. The hypothesis that we are left with is that children, teenagers and young adults that have more severe forms of the ASD—as those described in our current study and by Vilensky et al. (1981)—may be more likely to show differences in spatiotemporal parameters of gait, as well as postural differences and increases in aberrant movements (e.g., hand flapping, ballistic movements, etc.). Alternatively, individuals diagnosed as “high functioning Autism” or Asperger Syndrome—as described by Hallett et al. (1993); Rinehart et al. (2006a,b) and Calhoun et al. (2011)—will show less evident variations in spatiotemporal parameters, but meaningful variations in balance and posture contributing to these individuals qualitatively seeming “awkward” in their movement.

### Is there a relation between differences in gait and severe verbal communication disorders?

It was not surprising to us that we confirmed out hypothesis of widespread variations in the gait patterns of individuals diagnosed with severe forms of ASD and low Verbal Communication as compared to typically developing university undergraduate students. As indexed by the qualitative ratings in our study sample and as indicated by the very criteria of Autism in the DSM-IV-R (2000), movement disorders are rife within this population. However, our data raises the question of whether the differences in movement may be more acute in a group of young adults who present with profoundly low Verbal Communication.

What has been surprising to us is that there are so few questions being asked in the literature on ASD and movement about the “chicken and egg” aspects of the relation between disorders of movement, disorders of verbal expression and global ratings of “severe” forms of Autism (cf., Donnellan et al., 2010). Our study does not answer questions about the relation between different facets of movement in individuals diagnosed with ASD *per se*, other than to show that individuals with highly impaired verbal expression also have significantly different gait patterns compared to typically developing young adults. What this study should do is to raise further questions about what the interrelations among different aspects of movement dysregulation may be. Is there more than just a correlation between aberrant gait and inability to speak verbally? Or, may it be the case that aberrations in movement patterns can manifest in a variety of ways both across different individuals with the ASD diagnosis or among different movement systems for a single individual? We suspect that differences in walking have an analogous etiological and developmental trajectory as does the emergence of aberrations in speech and language.

## General conclusions

In comparing our data to that reported by others (Vilensky et al., 1981; Hallett et al., 1993; Vernazza-Martin et al., 2005; Rinehart et al., 2006a,b; Calhoun et al., 2011), it would appear that there are greater and more widespread differences in spatiotemporal parameters of gait among individuals who present with more severe forms of ASD and verbal communication disorders. Though the current study does not allow for more than a correlational coupling of gait and verbal expression, we believe the next steps in this research will require asking questions about the interrelation of movement systems within individuals. We need to consider the fundamentally circular etiological question of “which comes first,” disorders of communication and social interaction, or aberrations in movement? We propose that greater attention must be paid to hypotheses that Autism is primarily a disorder of movement first. This is clearly consistent with the neuro-anatomical and neuro-imaging data demonstrating significant aberrations of the cerebellum (Courchesne et al., 1999; Allen et al., 2004; Bauman and Kemper, 2005a,b) and the Basal Ganglia (Hollander et al., 2005; Langen et al., 2007). There is clear evidence that differences in the cerebellum must have gone awry during early embryological development (Bauman and Kemper, 1994, 2003, 2005a,b; Rodier et al., 1996; Rodier and Arndt, 2005). As such, disorders of verbal expression, disorders of social interaction and hence the global diagnosis of ASD may be secondary to the primary disorder—or core deficit in ASD—of developmental anomalies of movement. It is clearly time to advance questions that consider the etiology of ASD as it relates to the trajectory of movement across development.

### Conflict of interest statement

The authors declare that the research was conducted in the absence of any commercial or financial relationships that could be construed as a potential conflict of interest.
